# Iron Deficiency Screening in Patients Admitted with Acute Heart Failure Decompensation

**DOI:** 10.1007/s12265-026-10774-8

**Published:** 2026-05-19

**Authors:** Alex M. Sindledecker, Yassar Nabeel, Olivia Palladino, Lawrence A. Frazee

**Affiliations:** 1 Pharmacy - Cleveland Clinic Akron General, 1 Akron Gen Ave, Akron, OH 44307 USA; 2 Cardiovascular Medicine - Cleveland Clinic Akron General, 1 Akron Gen Ave, Akron, OH USA; 3 Medicine with Northeast Ohio Medical University, Rootstown, OH USA; 4 Ohio University Heritage College of Osteopathic Medicine, Athens, OH USA; 5 Clinical Associate Professor Pharmacy Practice, Northeast Ohio Medical University, Akron, OH USA

**Keywords:** Heart failure, Intravenous iron, Iron studies, Iron deficiency, Iron assessment

## Abstract

Correction of iron deficiency (ID) with intravenous iron among patients with heart failure (HF) may improve outcomes but is rarely assessed in real-world studies. Hospital admission for decompensated HF presents an opportunity to identify patients for treatment of ID. Of 2,275 patient admissions for decompensated HF, iron studies were available for 526 (23.1%) and ID was identified in 332 (63.1%) patients. Iron replacement was oral in 241 (10.6%) or intravenous in 285 (12.5%) with 132 (46.3%) patients receiving treatment during the admission. Anemia, chronic kidney disease stage 3 (CKD3), and low mean corpuscular volume (MCV) were associated with a higher odds of iron study availability while age was associated with lower odds. Frequency of assessment of ID in HF patients remains low. Predictors of iron status assessment indicate that factors related to anemia rather than HF are likely responsible for iron status assessment among these patients.

## Introduction

Heart failure (HF) is a syndrome that can impair quality of life (QoL), lead to hospital admissions, and increase mortality [1]. Although appropriate therapy for HF can improve these outcomes, HF decompensation is a leading cause of hospitalizations for patients age 65 years and older [2]. Additionally, the number of people in the United States with HF is expected to exceed 8 million individuals by 2030 at an estimated annual cost of $70 billion, most of which is for inpatient care [3]. Interventions that improve outcomes and reduce hospitalizations will have a significant human and economic impact.

Iron deficiency (ID) has been associated with worse outcomes and more frequent hospitalizations in patients with HF [4, 5]. The prevalence of ID in this population is also estimated to be 30–50% in stable HF patients and up to 80% in those with acute decompensated heart failure (ADHF) with or without anemia [6–8]. Oral iron replacement did not improve exercise capacity compared to placebo in patients with heart failure with reduced ejection fraction (HFrEF) [9]. Intravenous iron replacement improved functional capacity, heart failure symptoms, and QoL [10–12] and reduced hospitalizations for heart failure [13]. None of these studies planned for an assessment of mortality or acute decompensation. Cardiovascular mortality as part of a composite end-point was reduced in one clinical trial [14]. Recent clinical trials were not able to demonstrate a difference in cardiovascular mortality [5, 15]. Meta-analyses have demonstrated a reduction in all-cause mortality or cardiovascular hospitalization as a composite outcome [16–18]. A recent meta-analysis of clinical trials of IV iron in HF patients with ID demonstrated a lower rate of the composite outcome, recurrent HF hospitalizations and cardiovascular mortality [19].

Major Cardiology Societies have recommended assessing and treating ID in patients with HF. The 2022 AHA/ACC/HFSA guideline for the management of heart failure recommends intravenous iron in patients with HFrEF with or without anemia to improve functional status and QoL as a Class IIa recommendation [20]. The European Society of Cardiology guidelines for the diagnosis and treatment of acute and chronic heart failure give a Class I recommendation for the use of intravenous iron in symptomatic HFrEF patients and a Class IIa recommendation for heart failure with mid-range ejection fraction (HFmrEF) [21, 22].

Despite guideline recommendations, the frequency of iron status assessment generally appears to be low in real world practice [23, 24]. Currently, it is unknown how often iron status is assessed or known at the time of admission for ADHF, or which patients are more likely to have iron studies available and receive treatment. This study sought to determine the frequency and predictors of iron status assessment among patients admitted for ADHF. This information will reveal opportunities for treatment of ID during a hospital admission when patients could receive intravenous iron therapy.

## Methods

### Study Design

This was a retrospective study of patients admitted to a Cleveland Clinic Regional Hospital between July 1, 2021, and June 30, 2024, with a principal admitting diagnosis of ADHF. The protocol was approved by the Cleveland Clinic Institutional Review Board.

### Patient Selection

All patients admitted to a Cleveland Clinic Regional Hospital between July 1, 2021, and June 30, 2024, with a principal admitting diagnosis of ADHF were identified. Reasons for exclusion were age less than 18 years, end stage renal disease (ESRD), chronic kidney disease stage 4 (CKD4), or pregnancy at any time during the study period.

### Patient Characteristics

Patient characteristics collected include age, sex, race, medical comorbidities, body mass index (BMI), LVEF, hemoglobin (Hgb), mean corpuscular volume (MCV), and diagnosis of gastrointestinal (GI) bleed on admission. Variables reported as present on admission (Hgb, MCV, and BMI) were accepted if resulted between ± 1 day from the date of admission. LVEF was reported as the most recent result within 180 days prior to the time of admission or during admission.

### Outcomes

Iron study components, ferritin and transferrin saturation (TSAT) were reported as the most recent result within 180 days prior to the time of admission or during the admission. Iron study availability was defined as the presence of both a ferritin and a TSAT within this timeframe. Frequency of availability of iron status is reported for all admissions as well as the first (index) admission for each unique patient within the study period. The specific time period was selected to allow an adequate number of patients admitted before and after publication of AHA/ACC/HFSA guidelines [20] formally recommending iron assessment and treatment. Iron deficiency was defined and reported using both the standard guideline definition (ferritin < 100 mcg/L or ferritin 100–299 mcg/L and TSAT *≤* 20%) and a modified definition (ferritin < 100 mcg/L or TSAT < 20%). Predictors of iron study availability were collected for the index admission in the time period for each unique patient and included age, active GI bleed on admission, anemia (defined as a hemoglobin < 13 in males and < 11.5 in females), low MCV (defined as a MCV < 80 fL) on admission, not admitted to a cardiology service, female sex, chronic kidney disease stage 3 (CKD3), LVEF *≤* 40%, and admission date after publication of the AHA/ACC/HFSA guidelines. Patients were considered to be receiving oral iron supplementation if an oral iron supplement was on the patient’s medication list within 90 days prior to the admission date. Intravenous iron supplementation was determined by an order for an intravenous iron product 6 months before admission, during admission, or 6 months after the admission date.

### Statistical Analysis

For the primary objective, availability of iron studies is presented as the prevalence of all index ADHF admissions for which iron studies were available. Whether the iron studies resulted prior to or during the admission is also presented. A multiple logistic regression model was fit to assess the predictors of iron study availability and presented as an Adjusted Odds Ratio (aOR) with 95% Confidence Interval. Analysis was performed using SAS Software (Version 9.4, Cary, NC). For the secondary objective, the prevalence of ID among index ADHF admissions for which iron studies are available is presented. Iron treatment regimens among all patients are presented as number of patients (n) with proportion (%) of all patients included.

## Results

### Patient Characteristics

**Fig. 1:**
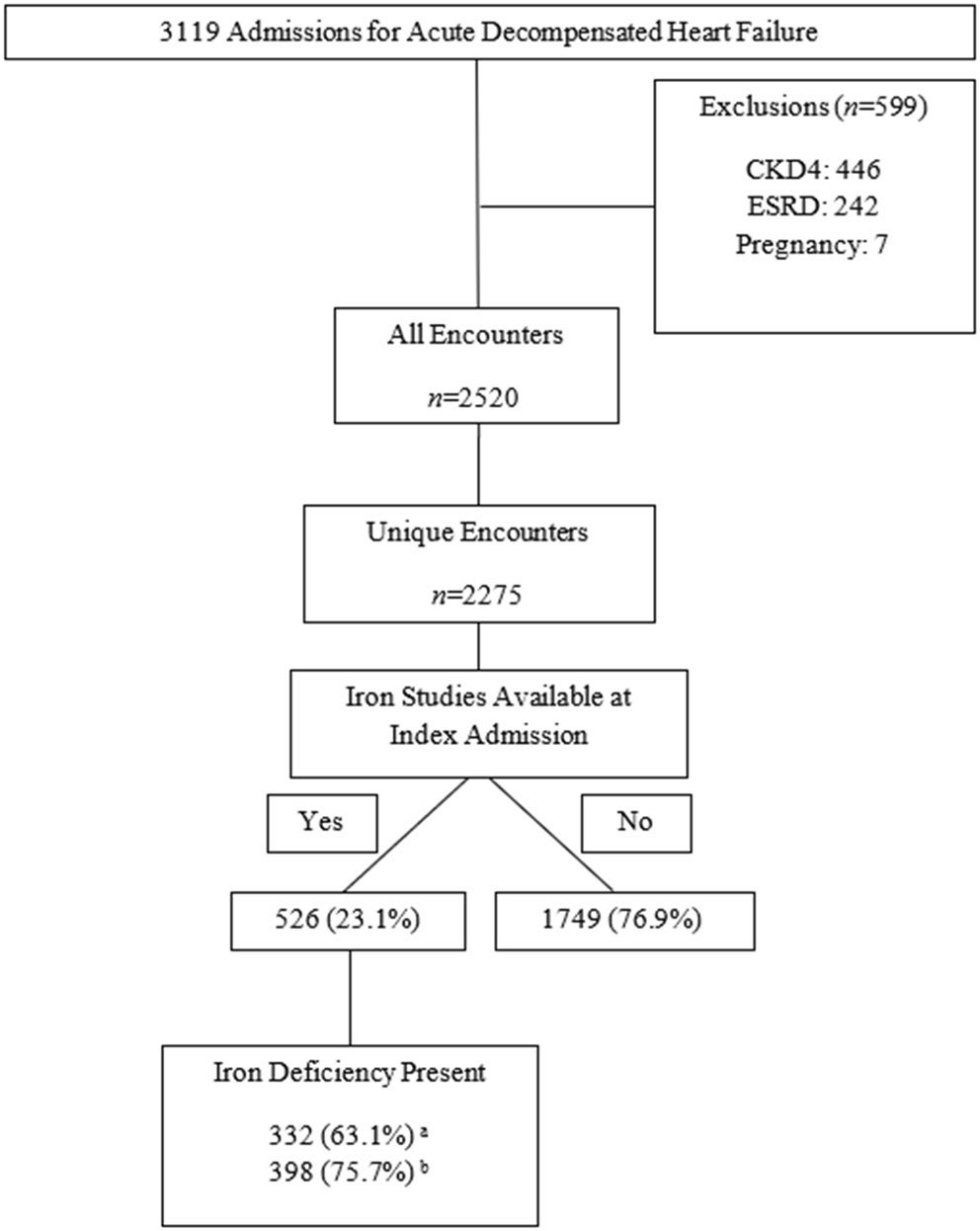
Study flow diagram. Patients may have met more than one exclusion criteria. ^**a**^Guideline Definition -- ferritin <100 mcg/L, or ferritin of 100-299 mcg/L and TSAT of <20% [19], [21], [32], ^**b**^Modified Definition -- ferritin <100 mcg/L and/or TSAT <20% [11], [29]

A total of 3119 encounters were reviewed for inclusion. After screening, 599 encounters were excluded with the most common reason being CKD4 (*n* = 446) and ESRD (*n* = 242). Patients may have met more than one exclusion critieria. After exclusion, 2520 encounters with 2275 unique index admission encounters were included for analysis (Fig. 1). The study population was mostly white (75.7%), male (54.8%) with a median age of 76 years (IQR 65-85). Type 2 diabetes (38.7%), CKD3 (28.3%), and coronary artery disease (37.8%) were the most prevalent comorbidities, and most patients were either overweight (32.6%) or obese (59.0%). When classifying LVEF, most encounters had a reported LVEF of ≥ 50% (49.8%) with LVEF ≤ 40% (41.3%) as a close second. See Table 1.

**Table 1:** Baseline characteristics

Characteristic	*n* = 2275	
Age – yr		
Median (IQR)	76	(65-85)
Sex – no. (%)		
Male	1246	(54.8)
Female	1029	(45.2)
Race – no. (%)		
Asian	14	(0.6)
Black	461	(20.3)
Other	78	(3.4)
White	1722	(75.7)
Comorbidities – no. (%)		
Type 1 Diabetes Mellitus	44	(1.9)
Type 2 Diabetes Mellitus	881	(38.7)
Hypertension	168	(7.4)
Chronic Kidney Disease Stage 1	2	(0.1)
Chronic Kidney Disease Stage 2	27	(1.2)
Chronic Kidney Disease Stage 3	644	(28.3)
Coronary Artery Disease	860	(37.8)
Ischemic Heart Disease	32	(1.4)
Overweight (*n* = 1360)	444	(32.6)
Obesity (*n* = 1360)	802	(59.0)
Left Ventricular Ejection Fraction – no. (%) (*n* = 1986)		
LVEF ≤ 40%	821	(41.3)
LVEF 41–49%	175	(8.8)
LVEF ≥ 50%	990	(49.8)
Hemoglobin on admission – mean (SD)	11.9	(2.2)
Gastrointestinal bleeding upon/during admission – no. (%)	14	(0.6)

Baseline demographics for the study population. Included characteristics that may have influence on outcomes within this study

### Outcomes

For all encounters (*n* = 2520) that met inclusion criteria, 562 (22.3%) had iron studies available within the previous 6 months or during admission. For the analyzed index admissions (*n* = 2275) iron studies were available in 526 (23.1%) encounters. Of the total 526 with iron studies available, 241 (45.8%) resulted during the admission and 285 (54.2%) resulted within 6 months prior to admission (Table 2). For the secondary outcome of iron deficiency among those who had iron studies available during the index admission, 332 (63.1%) were iron deficient by the current guideline definition and 398 (75.7%) were iron deficient by the secondary definition (Table 2). Each component of iron studies available is also summarized in Table 3. Both ferritin and TSAT were assessed at a similar frequency; 28.5% and 26.2% respectively. The ferritin level was < 100 ng/mL for 12.4% of index encounters and was between 100 and 200 ng/mL for 9.3% of encounters. TSAT was < 20% for 18.2% of encounters.

**Table 2:** Primary and secondary outcomes

	*n* = 2275	
Primary outcome: iron studies available – no. (%)	526	(23.1)
Iron studies obtained during admission – no. (%)	241	(45.8)
Iron studies obtained during previous six months – no. (%)	285	(54.2)
Iron deficiency present among patients with iron studies available – no. (%)	*n* = 526	
By guideline definition^a^	332	(63.1)
By modified definition^b^	398	(75.7)

^a^ferritin < 100 mcg/L, or ferritin of 100–299 mcg/L and TSAT of < 20% 20, 22, 32

^b^ferritin < 100 mcg/L and/or TSAT ≤ 20% 14, 23

**Table 3:** Components of iron studies

	*n* = 2275	
Ferritin		
Ferritin assessed – no. (%)	648	(28.5)
Ferritin – median (IQR)	119.8	(52.9-289.6)
Ferritin < 100 – no. (%)	282	(12.4)
Ferritin 100 to 299 – no. (%)	212	(9.3)
TSAT		
TSAT assessed – no. (%)	596	(26.2)
TSAT – median (IQR)	13.7	(8.6–23.0)
TSAT < 20 – no. (%)	414	(18.2)
Ferritin and TSAT		
Ferritin and TSAT assessed – no. (%)	526	(23.1)
Iron		
Iron – median (IQR)	37	(25.8–56.0)
Iron < 13 – no. (%)	15	(0.7)
TIBC		
TIBC – mean (SD)	285.5	(80.3)

The components that make up iron studies are listed above. To meet the iron deficiency criteria, only ferritin and TSAT values are required. Both iron and TIBC are reported out for completeness as they are included on iron panels at our institution

Iron treatment regimens are summarized in Table 4. Oral iron treatment was utilized in 241 (10.6%) encounters whereas intravenous iron was utilized in 285 (12.5%) encounters. Of the 285 patients who received intravenous iron, almost half received it during the admission (*n* = 132, 46.3%) while the rest received it at some time outside the admission (either outpatient or at a different admission). Ferric gluconate was the most common intravenous iron product administered with iron sucrose, being the second most common.

**Table 4:** Iron treatment regimens

Iron treatment regimen – no. (%)	*n* = 2275	
Oral iron	241	(10.6)
Intravenous iron	285	(12.5)
Inpatient intravenous iron - no. (%)	132	(46.3)
Intravenous iron product received^a^	*n* = 285	
Ferric gluconate	236	(82.8)
Iron sucrose	66	(23.2)
Ferric derisomaltose	9	(3.2)
Iron dextran	8	(2.8)
Ferric carboxymaltose	6	(2.1)
Ferumoxytol	0	(0.0)

^a^Some patients received more than one intravenous iron product. Among the 285 patients that received intravenous iron, there were 325 total administrations of an intravenous iron product

### Predictors of Iron Study Availability

Predictors of iron study availability are presented in Table 5. Age, anemia, CKD3, and MCV less than 80 fL were statistically significant predictors of iron availability in a multiple logistic regression analysis. For every 10-year increase in age, the odds of iron study availability decreased by 11% (aOR 0.89 (0.83–0.97)). Those with anemia, CKD3, and MCV less than 80 fL had higher odds of iron study availability (5.74 (4.21–7.83); 1.70 (1.36–2.12); and 2.04 (1.45–2.86), respectively)).

**Table 5:** Predictors of iron studies - multivariable logistic regression

Effect	Adjust Odds Ratio (95% CI)
Age (Decades)	0.89 (0.83–0.97)
Active GI^a^ bleed on admission	1.61 (0.46–5.71)
Anemia	5.74 (4.21–7.83)
Admitted to cardiology	0.94 (0.54–1.63)
CKD3^b^ on admission	1.70 (1.36–2.12)
Female	0.90 (0.73–1.12)
MCV^c^ less than 80 on admission	2.04 (1.45–2.86)
Admitted post guideline update	0.88 (0.71–1.09)
LVEF^d^ ≤40%	1.19 (0.94–1.50)

CI = Confidence intervals. Note subjects that were missing one or more of the predictor variables were excluded from the model

^a^gastrointestinal; ^b^chronic kidney disease stage 3; ^c^mean corpuscular volume; ^d^left ventricular ejection fraction

## Discussion

This was a retrospective study to assess availability and predictors of availability of iron studies among patients at the time of admission for ADHF. The prevalence of ID and iron treatment is also described. In this study, assessment of iron status (23.1%) was lower than previously described in a large registry database review looking at iron status assessment over time and in three different calendar years [24], but similar to what was reported in another earlier registry report where assessment rates were < 25% in 2018 [25]. In addition, nearly half of the iron assessments that reported both a ferritin and TSAT (45.8%) resulted during a hospital admission. The CARENFER study reported that centers surveyed in France were more likely to assess iron status during an admission (80%) as compared to prior to admission (7.7%) [23].

Guidelines define ID as serum ferritin of < 100 mcg/L, or a serum ferritin of 100–299 mcg/L and a TSAT of < 20%. However, the use of ferritin to define ID in HF has recently been called into question [26] as there is some evidence that a lower ferritin by itself is associated with better heart failure outcomes [27–29]. A revised definition of ID (ferritin < 100 mcg/L and/or TSAT *≤* 20%) would increase the number of patients defined as iron deficient [30]. TSAT decreases in inflammatory states since it is not being “saturated” with iron and use of a TSAT *≤* 20% alone may be a more accurate marker of ID in HF [31]. To illustrate this, analysis of the IRONMAN trial revealed that TSAT > 20% and lack of anemia were associated with the least benefit from intravenous iron, whereas anemia and TSAT *≤* 20% with a ferritin > 100 mcg/L were associated with the most benefit. In CARENFER, classification of ID increased from 49.6% to 62.8% of patients with heart failure using the modified definition [23]. In this study, ID was assessed based on the guideline definition as well as the modified definition and demonstrated that classification as iron deficient increased from 63.1% to 75.7%, respectively.

Minasian et al. reported that during the years 2012, 2017, and 2022 administration of intravenous iron for those who were iron deficient was 4%, 11%, and 14% respectively [24]. In this study, 9.4% of the population received an intravenous iron product within ± 6 months of the admission and nearly half (45.7%) received it during the admission. In this study, the attempt to connect iron administration with a diagnosis of ID was not made, but assumed that intravenous iron would only be given if ID was diagnosed. If this is the case, then approximately half of the patients in this study identified as iron deficient by the guideline definition received intravenous iron. Whether they received an appropriate dose or were reassessed for further intravenous iron is unknown.

Predictors of iron status assessment were evaluated. It was assumed that anemia, microcytosis, gastrointestinal bleeding, admission to a cardiologist service, admission after publication of the AHA/ACC/HFSA guidelines, and reduced LVEF would be more likely to result in iron status assessment. It has been reported that ID is more prevalent in patients with an LVEF ≥ 50% [23], but there is currently no evidence that treating ID in these patients is beneficial. Patients with CKD4 and ESRD were excluded from this study, considering that anemia of advanced chronic kidney disease involves careful consideration of iron status and replacement. As hypothesized, anemia and a low MCV were independently associated with a higher odds of iron status assessment. Combined with the observation that a reduced LVEF and admission to a cardiology service were not associated with a higher odds of iron status assessment, it is believed that much of the iron status assessment in this study was related to evaluation for microcytic anemia and not HF, specifically. Additionally, the presence of CKD3 was associated with a higher odds of iron status assessment, likely indicating an evaluation for anemia in these patients. Lastly, admission after publication of the AHA/ACC/HFSA guideline update in 2022 was not associated with a higher odds of iron status assessment, further strengthening the conclusion that iron status assessment was likely not related to HF management.

This study had several limitations. First, this was a retrospective review of electronic medical records over a 3-year period and so was dependent on medical record accuracy and documentation in a real-world clinical setting. For inclusion criteria, all patients admitted with a principal diagnosis of ADHF were based on ICD10 codes and an attempt to confirm this via manual chart review was not conducted. It is possible that admissions where the reason for admission ended up not being ADHF were included. Also, to avoid confounding from patients with advanced kidney disease, patients diagnosed with CKD4 or with ESRD at any time in the study period were excluded. Second, there was an inability to accurately acquire prior to admission medications to assess the presence of HF medications. Prescriptions for medications from providers outside the system could not be accurately captured. As such, there was no ability to assess the prevalence of medications (sodium-glucose transport 2 inhibitors, renin-angiotensin-aldosterone inhibitors, and neprilysin inhibitors) that may alter iron studies and CKD classification [31]. Oral iron use may have been under- or over-reported since a 90-day prescription criterion was used and it was not possible to know if the patient received the medication. Since intravenous iron must be administered in a health care setting, it is believed that all intravenous iron received was accurately captured. It is possible that patients received intravenous iron through a different health system, which would not have been captured in our study. Next, assessment of certain characteristics was dependent on their presence at a particular time relative to the admission date. Hgb and MCV were determined on the day of admission (± 1 day) to tie it to that admission specifically. Finally, categorization of patients by LVEF was based on the most recent LVEF available within the prior 6 months. A small number of patients (12.7%) did not have an LVEF available and had to be excluded from the multivariable model. The most recent LVEF may not reflect a diagnosis of reduced or preserved LVEF as some of those patients may have been diagnosed as reduced LVEF that subsequently improved.

## Clinical Relevance

In summary, guidelines recommend assessment for ID and iron replacement in patients with HF, especially with reduced LVEF. There has been a low uptake of this recommendation and inpatient admission for ADHF may be an opportunity to capture these patients. This retrospective study demonstrated a low rate of iron status assessment for patients during an admission with ADHF, similar to previous real-world studies of outpatients with HF. Intravenous iron was used in a minority of patients and was administered during the admission for ADHF in nearly half of them. Predictors of iron status assessment indicate that factors related to anemia rather than HF are likely responsible for iron status assessment among these patients. This illustrates the need to develop a systematic approach to assess iron status and treat ID in HF patients where appropriate. Future studies should report on implementation of a systematic approach to assess and treat ID in patients with HF.

## Abbreviations

ACC: American College of Cardiology
ADHF: Acute decompensated heart failure
AHA: American Heart Association
aOR: adjusted odds ratio
BMI: body mass index
CI: confidence interval
CKD: chronic kidney disease
ESC: European Society of Cardiology
ESRD: end stage renal disease
GI: gastrointestinal
Hgb: hemoglobin
HF: heart failure
HFmrEF: heart failure with mid–range ejection fraction
HFrEF: heart failure with reduced ejection fraction
HFSA: Heart Failure Society of America
ID: iron deficiency
IQR: interquartile range
LVEF: left ventricular ejection fraction
MCV: mean corpuscular volume
QoL: quality of life
SD: standard deviation
SGLT2i: sodium–glucose cotransporter–2 inhibitor
TSAT: transferrin saturation
